# Cellular Mechanisms Underlying the Cardioprotective Role of Allicin on Cardiovascular Diseases

**DOI:** 10.3390/ijms23169082

**Published:** 2022-08-13

**Authors:** José L. Sánchez-Gloria, Abraham S. Arellano-Buendía, Juan G. Juárez-Rojas, Fernando E. García-Arroyo, Raúl Argüello-García, Fausto Sánchez-Muñoz, Laura G. Sánchez-Lozada, Horacio Osorio-Alonso

**Affiliations:** 1 Sección de Estudios de Posgrado, Escuela Superior de Medicina, Instituto Politécnico Nacional, Mexico City 11340, Mexico; 2 Department of Immunology, Instituto Nacional de Cardiología Ignacio Chávez, Mexico City 14080, Mexico; 3 Department of Cardio-Renal Physiopathology, Instituto Nacional de Cardiología Ignacio Chávez, Mexico City 14080, Mexico; 4 Department of Endocrinology, Instituto Nacional de Cardiología Ignacio Chávez, Mexico City 14080, Mexico; 5 Departamento de Genética y Biología Molecular, Centro de Investigación y de Estudios Avanzados del IPN, Mexico City 07360, Mexico

**Keywords:** allicin, cardiovascular disease, risk factors, dyslipidemia, hypertension, hypertrophy, inflammation, oxidative stress, endothelial dysfunction

## Abstract

Cardiovascular diseases (CVDs) are a group of diseases in which the common denominator is the affection of blood vessels, heart tissue, and heart rhythm. The genesis of CVD is complex and multifactorial; therefore, approaches are often based on multidisciplinary management and more than one drug is used to achieve the optimal control of risk factors (dyslipidemia, hypertension, hypertrophy, oxidative stress, endothelial dysfunction, inflammation). In this context, allicin, a sulfur compound naturally derived from garlic, has shown beneficial effects on several cardiovascular risk factors through the modulation of cellular mechanisms and signaling pathways. Effective pharmacological treatments for CVD or its risk factors have not been developed or are unknown in clinical practice. Thus, this work aimed to review the cellular mechanisms through which allicin exerts its therapeutic effects and to show why it could be a therapeutic option for the prevention or treatment of CVD and its risk factors.

## 1. Introduction

The cardiovascular system (CS) supplies oxygen and nutrients to cells and tissues of the body and, it also removes metabolic waste products. CS includes heart and blood vessels (veins, arteries, arterioles, and capillaries). Alterations in the structure or function of CS components lead to the development of a group of diseases that affect blood vessels, heart, and heart rhythm. Because these diseases share several characteristics concerning their onset and pathophysiology, as well as prognosis and treatment, these entities are collectively denominated as cardiovascular diseases (CVDs) [1].

Nowadays, CVD is one of the major causes of morbidity and mortality both at the regional level and worldwide [2,3]. Moreover, several studies have predicted an increase in the incidence for years to come [2,3,4]. In Latin America and Mexico, CVDs are the first cause of mortality (20.8%), followed by COVID-19 (15%) and diabetes mellitus (14.6%), without significant differences associated with gender [1,4,5]. The World Health Organization (WHO) reported that heart diseases are the leading cause of mortality for the last 20 years, reaching 19 million deaths in 2019. This significant death toll represents 16% of deaths from all other causes [2,3,6].

**Figure 1:**
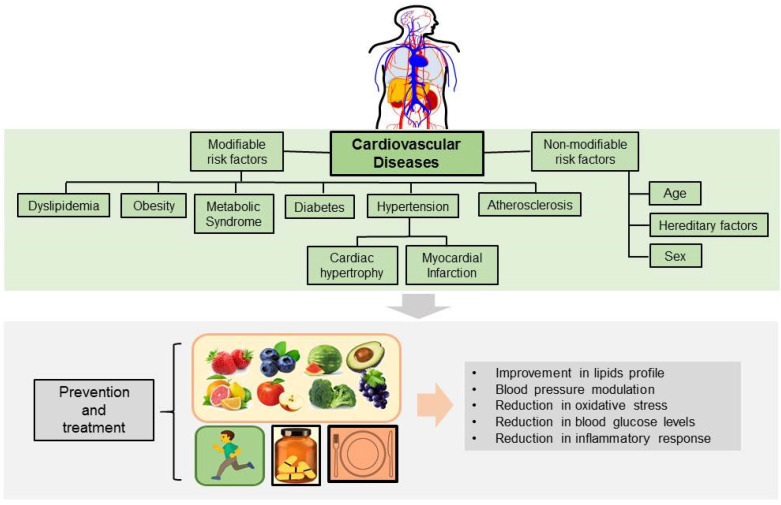
Cardiovascular diseases: classification of risk factors, physiological effects and strategies for prevention and treatment.

The pathogenesis of CVD is multifactorial, complex, and progressive, and develops due to the interaction of several factors (environmental, dietary, race, and metabolic), secondary to other diseases (hypertension, diabetes, metabolic syndrome, etc.), and may also be of genetic origin, or caused by birth defects [7,8]. CVDs with genetic or congenital background represent a lower percentage (30%) as a cause, compared to CVDs originated by the interaction of other factors (70%) [9]. The factors associated with the pathogenesis of CVD are known as risk factors) and have been classified as modifiable and non-modifiable. The modifiable risk factors are also called susceptible to modification or control and include hypertension, dyslipidemia, metabolic syndrome, diabetes, obesity, smoking, alcoholism, diet, and sedentary lifestyle. The non-modifiable risk factors include age, sex, and ethnic or hereditary factors (Figure 1) [9,10,11].

CVD includes entities that affect the entire circulatory system (Figure 1); thus, different organs and tissues are affected and consequently, there are various clinical subtypes, based on the injured tissue as well as the symptoms/signs manifestation (Table 1) [1,2,7,8,9]. CVD develops early in life and progresses slowly, so it often progresses unnoticed for a long time before the first symptoms become clinically apparent. Chronic exposure to risk factors promotes cellular and biochemical responses, as well as intracellular defense mechanisms that trigger reactive oxygen species (ROS) formation, endothelial dysfunction, inflammation, fibrosis, apoptosis, vascular remodeling, and hypertrophy. These alterations induce tissue remodeling and dysfunction that if left untreated, progress to advanced stages.

Besides being one of the main causes of mortality worldwide, CVDs cause the disbursement of significant amounts of money for the management of the disease in the short, medium, and long term. The treatment of CVD is primarily focused on the control of modifiable risk factors [12,13], combining pharmacological treatment and changes in lifestyle [14,15]. However, due to the complexity of CVD pathogenesis, the treatments are usually approached with multidisciplinary management, and more than one drug is used to achieve the optimal control of risk factors. Despite the government’s efforts, the incidence of CVDs continues as high as before and their incidence is still far from decreasing.

One of the non-pharmacological therapeutic strategies to reduce risk factors involves changes in lifestyle (modifying eating habits and physical activities) (Figure 1). On the other hand, proactive functional medicine is a growing strategy that displays not only health-promoting activities but therapeutic potential as well [16,17,18]. In this context, preclinical and clinical studies have reported that the intake of vegetables and some types of fruits, herbs, and spices provide elements and micronutrients with cardioprotective properties [18]. The main properties that have been described for nutrients include anti-inflammatory, anti-diabetic, anti-hypertensive, and anti-oxidant activities (Figure 1) [18,19,20]. Furthermore, it is well known that many drugs have been obtained or derived from plants, vegetables, fruits or other natural origins [19,21].

Garlic is a spice commonly used as a condiment, but since ancient times activities such as anti-microbial (parasites, bacteria, and fungi), anti-diabetic, anti-hypertensive, and cardioprotective have been reported [22]. Garlic is especially rich in sulfur-containing compounds; thus, many of these compounds can be responsible for its therapeutic effects. Recent studies have shown that allicin, a garlic-derived sulfur compound, has beneficial effects on different cell types that could be useful for the management of CVD or its risk factors. Therefore, in the present work, we review the preclinical and clinical studies and summarize the cellular and molecular mechanisms by which allicin exerts its beneficial activities and place it as a potential therapeutic option in the treatment of CVD.

## 2. Allicin

### 2.1. Garlic as a Natural Source of Allicin

Garlic is useful in the treatment of CVD, mainly due to its anti-inflammatory, anti-hypertensive, anti-platelet, and anti-diabetic effects [21,22,23,24,25]. However, recent studies suggest that the biological activities in garlic can be attributed to allicin, the compound formed in high proportion when a garlic clove is cut, macerated, or crushed. Therefore, it has been hypothesized that allicin is the main compound responsible for the beneficial effects of garlic consumption.

Raw garlic water content is approximately 50%, the rest consists of carbohydrates, lipids, proteins, fiber, vitamins, free amino acids, and minerals, and it is especially rich in sulfur compounds (Table 2) [22,23,24,26].

**Figure 2:**
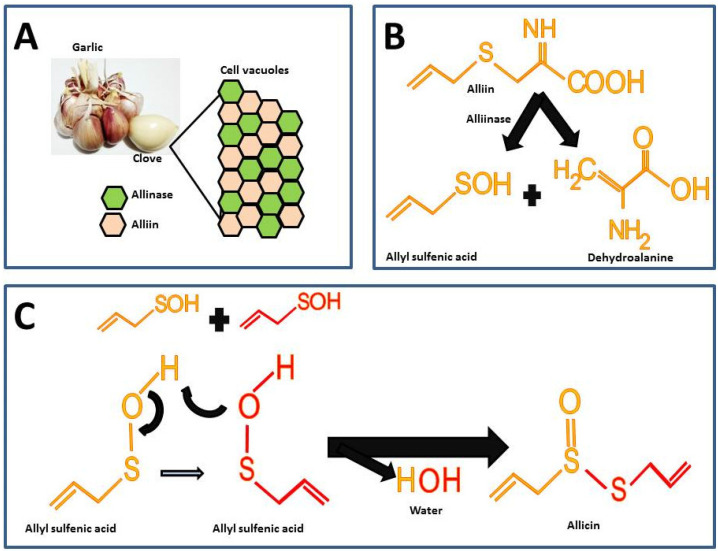
Chemical reactions for allicin synthesis in crushed garlic cloves and substrate formation for chemical synthesis: (**A**) Compartmentalization of alliin and alliinase in the intact garlic bulb; (**B**) First reaction in allicin formation; (**C**) Second reaction for the allicin formation.

Garlic is odorless until the alliin compound [(+)-S-(2-propenyl)-L-cysteine sulfoxide] and the alliinase enzyme react. These components in intact garlic bulbs are stored in vesicles or vacuoles independently until mechanical stimuli such as cutting or maceration break it, thus, alliin and alliinase are released and the catalysis for allicin formation takes place (Figure 2A) [26]. There are other sulfur compounds in raw garlic, but the proportion of these is lower compared to the alliin.

The highest percentage of sulfur compounds in garlic is represented by γ-L-glutamyl-S-(2-propenyl)-L-cysteine (GSAC) an alliin precursor, which is transformed into alliin the substrate in the allicin synthesis reaction [26]. For the synthesis of allicin, alliin is hydrolyzed by the enzyme alliinase into allyl sulfenic acid and dehydroalanine (Figure 2B) [27]. Allicin is formed by the reaction from two allyl sulfenic acid molecules, resulting in allicin and one water molecule as a final product (Figure 2C).

The allicin synthesized represents 60 to 80% of all sulfur compounds (Table 2) and it gives the characteristic odor and hence is considered the active compound in garlic [24]. Furthermore, in spite of the fact that alliin and allicin have similar chemical residues, alliin does not show the biological activity of allicin [28,29]. In fact, a recent study assessed the effect of alliin on experimental obesity resulting in modest beneficial effects [29]. The latter supports the hypothesis that allicin is the active compound responsible for the biological activities in garlic.

**Table 2:** Composition of raw garlic (Allium sativum).

Substance or Compound		Percent in 100 g Dry Weight
Water		50%
Carbohydrates		30%
Proteins		10%
	Alliinase	10 mg/gr
	Free amino acid	1.0%
Lipids		3.5%
Fiber		1.0%
Kilocalories		149 Kcal
Vitamins		
	B1	0.16 mg
	B2	0.02 mg
	B6	0.32 mg
	Nicotinic Acid	0.12 mg
	Ascorbic Acid	14 mg
Minerals		
	Potassium	446 mg
	Phosphorous	134 mg
	Sodium	19 mg
	Calcium	17 mg
	Iron	1.2 mg
	Magnesium	24.1 mg
	Zinc	1.1 mg
	Iodine	4.7 µg
	Selenium	2 µg
Sulfur compounds		3.5%
γ-glutamyl peptides: 80–85%		
	γ-L-glutamyl-S-(2-propenyl)-L-cysteine (GSAC)	40–60%
	γ-L-glutamyl-S-(trans-1-propenil)-L-cysteine (GSPC)	10–18%
	γ-L-glutamyl-S-methyl-L-cysteine (GSMC)	10–18%
Sulfoxides produced by the allinase action:		
	(+)-S-(2-propenyl)-L-cysteine sulfoxide (alliin)	60–80%
	(+)-S-(trans-1-propenil)-L-cysteine sulfoxide (isoalline)	
	(+)-S-methyl-L-cysteine sulfoxide (methiine)	
	(1S, 3R, 5S) -5-methyl-1, 4-thiazan-3-carboxylic acid 1-oxide (cycloaliine).	

The content of garlic compounds is approximately considering the available scientific literature [22,24,26,30,31,32].

### 2.2. Synthetic Allicin

Allicin can also be obtained by chemical synthesis by a reverse process from the garlic decomposition cascade of natural synthesis. Briefly, diallyl disulfide dissolved in acetic acid is mixed with hydrogen peroxide to form allyl sulfenic acid, which, as described above, is the substrate for spontaneous condensation producing allicin (Figure 2C) that is further extracted from water with a polar organic solvent as dichloromethane. Through this synthetic procedure, allicin can be obtained with a high purity ranging from 90–92% [33]. Due to its hydrophobic nature, allicin can easily diffuse the phospholipid bilayer in cell membranes. Furthermore, it is known that allicin rapidly reacts with the free thiol or sulfhydryl groups of amino acids such as cysteine and the proteins on the cell membrane, as well as with other proteins in the cell compartments [34,35]. These characteristics could be important for the induction of the biological activities attributed to allicin.

## 3. Effects of Allicin on Cardiovascular Risk Factors

### 3.1. Dyslipidemia and Obesity

Atherogenic dyslipidemia [High levels of triglycerides (HTG), low high-density lipoprotein cholesterol (HDL-C), and high proportion of small and low-density lipoprotein cholesterol (LDL-C)], and obesity are two risk factors closely associated with the progression of CVD. Therefore, the control of both CVRF is one of the most important goals to achieve in the management of CVD, even in the premature stages [15]. In this regard, preclinical and clinical studies have shown that allicin has important beneficial effects on dyslipidemia and obesity. In this sense, in experimental models of hyperlipidemia, allicin improved the lipid profile (decreased TG, total cholesterol (TC), and LDL-C), and decreased hyperinsulinemia. In contrast, the allicin treatment increases HDL-C [36,37,38,39,40]. Additionally, other studies in hyperlipidemic rats reported that allicin prevented increased body weight and blood pressure [41,42]. Interestingly, in patients with type 2 diabetes, allicin reduced TC, and LDL-C, while HDL-C values were increased [25]. Moreover, in healthy subjects, the administration of allicin for 10 weeks reduced the concentrations of TC and TG [43,44]. In light of these observations, the beneficial effects of allicin on the lipid profile in dyslipidemia are convincing.

On the other hand, dyslipidemia is associated with poor vascular function. In this regard, in experimental hyperlipidemia high fat diet-induced, allicin reduced TG, TC, LDL-C, as well as alanine aminotransferase (ALT), and aspartate aminotransferase (AST) activities, while serum nitric oxide (NO) levels and relaxation degrees of thoracic aorta rings were increased [45]. Allicin-fenofibrate combination showed synergistic effects on the regulation of blood lipids and the improvement of endothelial function [45].

Moreover, dysregulated lipid metabolism signaling has long been known to be pathogenic in several CVD and can be caused by alterations in the metabolism of lipids and carbohydrates. In this context, the beneficial effects of allicin involved the modulation of signaling pathways underlying the synthesis or degradation of lipids and carbohydrates. Thus, allicin increased the phosphorylation of adenosine monophosphate-activated protein kinase (AMPK), protein kinase A (PKA), and the AMP response element-binding protein (CREB) in a cell culture of hepatic lipotoxicity (HepG2) [46]. In contrast, allicin decreased the expression of the sterol regulatory element-binding protein 1 (SREBP-1) and the protein SREBP-2. These proteins play a key role in the regulation of the metabolism of cholesterol, fatty acid synthesis, and insulin resistance [46]. Additionally, in an experimental model of nonalcoholic fatty liver disease (NAFLD) allicin reduced the lipid accumulation in the liver. This effect was related to the increase in PPARα and FABP6 expressions and to the down-regulation of PPARγ and FABP4 [47]. These allicin-induced effects contributed to the reduction in TG and TC accumulation in the cells of models of dyslipidemia and NAFLD [46,47].

In an experimental model of obesity high fat diet-induced, the allicin treatment increased the expression of genes involved in lipolysis [hormone-sensitive lipase (HSL), adipose triglyceride lipase (ATGL), and lipoprotein lipase (LPL)], and insulin signaling pathway [insulin receptor substrate 1 (IRS-1) and IRS-2]. In contrast, allicin decreased the expression levels of genes involved in lipogenesis [SREBP1, acetyl-CoA carboxylase (ACC), fatty acid synthase (FASN), stearoyl-CoA desaturase-1 (SCD-1), and peroxisome proliferator-activated receptor γ (PPARγ)] [48]. Finally, the allicin treatment reduced body weight gain and fat accumulation (visceral and subcutaneous), and improved the glucose tolerance test, insulin response, and lipid profile [48].

On the other hand, the beneficial effects of allicin include the modulation of metabolic organs such as brown adipose tissue (BAT), which is described as energy-consuming fat. Thus, the activation of BAT has been proposed as a therapeutic option in obesity [49,50,51]. Further, thermogenesis and glucose homeostasis are associated with BAT [52,53]. In relation to this, UCP1, PGC1a and PRDM16 are genes involved in thermogenesis, while KLF15 stimulates the expression of UCP-1 [54]. In adipocyte cell culture, allicin increased the expression of uncoupling protein-1 (UCP1), PPARγ co-activator 1α (PGC1α), PR-domain containing 16 protein (PRDM16), and Krüppel-like factor 15 (KLF-15) in a concentration-dependent manner [54]. In mice with a high-fat diet, the allicin treatment induced a decrement in the hepatic accumulation of cholesterol and TG, and lower serum TC, LDL-C, TG, and free fatty acids [54]. It is possible that the browning effect induced by allicin favors lipolysis and lipid oxidation and consequently improvements in the lipid profile and eventually the reduction in body weight, this may be beneficial in diseases such as obesity, insulin resistance, which are associated with overweight and dyslipidemia.

In this line, the allicin treatment reduced body weight gain, fat accumulation (visceral and subcutaneous), improved glucose tolerance test and insulin response, and the lipid profile. Additionally, it augments whole-body energy expenditure by activation of BAT (increase in UCP1 and OXPHOS-related proteins (ATP5A and NDUFB8)), increase in thermogenic genes (PGC1α/β and CPT1α/β) and mitochondrial biogenic transcription factors (NRF1, NRF2, and TFAM). Another important effect of allicin was the beiging of subcutaneous white adipose tissue (WAT) (increase in CD137, TMEM26, and TBX1), as well as the increase in expression of fatty acid oxidation (Sirt1, PGC1α, CPT1, and CPT1β) and lipolysis related genes (PPARα, HSL, and ATGL) in the liver, while reducing the related with fatty acid synthesis (SREBP1, ACC, FASN, and PPARγ). All of these effects contribute to relieving hepatic steatosis and inflammation in an experimental model of obesity diet-induced [55].

In patients, a randomized, double-blind, placebo trial, reported that C-reactive protein, and systolic, diastolic, and mean blood pressures decreased in NAFLD patients treated with allicin 3 mg/day [56].

**Figure 3:**
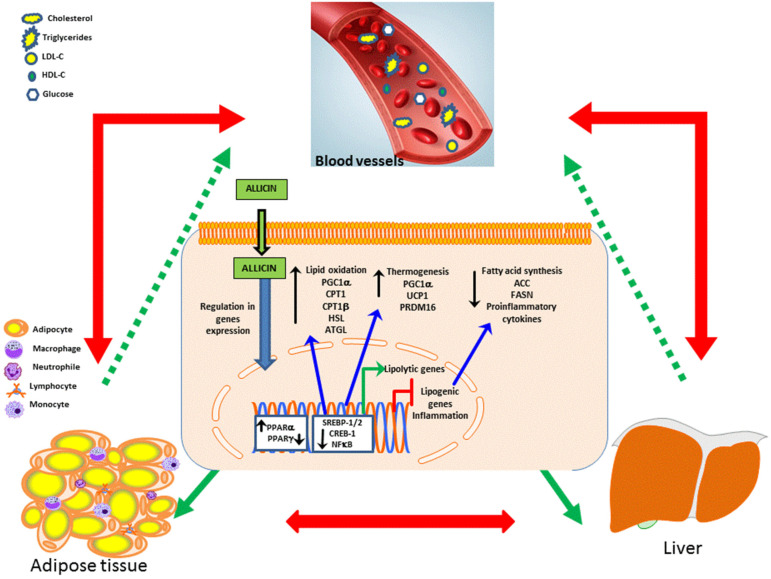
Effects of allicin on lipid metabolism: Allicin crosses the cell membrane and modulates transcription factors that regulate the expression of genes associated with lipid oxidation, thermogenesis, fatty acid synthesis and proinflammatory cytokines in the liver and adipose tissue improving the lipid profile. The red arrows indicate the close association between liver and adipose tissue dysfunction and its influence on the serum levels of the lipid profile and glycemia. The green arrows indicate the beneficial effects of allicin at the intracellular level in adipose and liver tissue and the final effect at the systemic level. Abbreviations: acetyl-CoA carboxylase (ACC), fatty acid synthase (FASN), AMP response element-binding protein (CREB); Sterol regulatory element-binding protein 1 (SREBP-1) and SREBP-2; Peroxisome proliferator-activated receptor alpha (PPARα); PPAR gamma (PPARγ); Pparγ coactivator 1α (Pgc1α); hormone-sensitive lipase (HSL); Adipose triglyceride lipase (ATGL); Uncoupling protein-1 (Ucp1); PR-domain containing 16 protein (Prdm16); low-density lipoprotein cholesterol (LDL-C); high-density lipoprotein cholesterol (HDL-C).

In summary, through the modulation of key proteins associated with the metabolism of lipids and carbohydrates allicin prevents body weight gain, maintains glucose homeostasis, and improves lipid profile, adiposity, and hepatic steatosis as observed in vitro and in vivo assays, as well as in patients with dyslipidemia (Figure 3). Therefore, allicin could contribute to the treatment of CVD through its effects on dyslipidemia, which is a common risk factor in obesity, metabolic syndrome, hepatic steatosis, and type 2 diabetes.

### 3.2. Atherosclerosis

Atherosclerosis is a gradual process of lipid accumulation, thickening, and hardening of arteries that occurs throughout a lifespan. These processes involve the formation of a plaque composed of lipids, fibrous tissue, and the activation and participation of inflammatory mechanisms (infiltration of cells and secretion of proinflammatory cytokines) [1]. Over time, plaques narrow arteries and that eventually will be destabilized releasing fragments of plaque that may block small vessels and capillaries interrupting the blood supply. The obstruction of arteries limits the flow of oxygen to the brain, heart, and lower extremities, increasing the risk of ischemic events [1]. HTG, hypercholesterolemia, and elevated levels of LDL-C and lipoprotein (a) (LPa), a specialized form of LDL-C, have shown to be the major triggers of atherosclerosis. In this context, it was demonstrated that allicin reduces the formation of fatty streaks and LDL-C oxidation in murine experimental models of atherosclerosis cholesterol-rich diet-induced [57,58]. Additionally, allicin decreased TG, TC, very-low-density lipoprotein cholesterol (VLDL-C), and LDL-C, while the HDL-C values were increased [58]. The beneficial effects of allicin on atherosclerosis were mediated by their antioxidant effects, lipoprotein modification, and the inhibition of LDL-C uptake and degradation by macrophages [58]. In addition, another protective effect of allicin is related to the modulation of inducible nitric oxide synthase (iNOS) expression in the cell culture of macrophages, iNOS promotes peroxynitrite formation and is expressed in atherosclerotic injury in humans [59]. Furthermore, allicin reduced hyperhomocysteinemia, TG, TC, and the intima-media thickness (IMT) of the carotid artery which is a marker of systemic atherosclerosis used in experimental models and in patients with coronary artery disease (CAD) [44,60].

Another study reported that allicin ameliorates experimental atherosclerosis, through modulation of gut microbiota and trimethylamine-N-oxide (TMAO) [61]. In healthy volunteers, allicin reduced TMAO in plasma and urine. The increase in blood TMAO has been associated with increased major adverse cardiovascular events and all-cause mortality [62,63].

On the other hand, vascular calcification (VC) affects arteries and valves of the heart and includes intimal and medial calcification in arteries. VC augments the risk for adverse cardiovascular events and is associated with atherosclerosis, obesity, diabetes, metabolic syndrome, hypertension, and mainly chronic kidney disease (CKD) and end-stage renal diseases (ESRD) [64]. In this context, allicin reduced the expression of osteoblast markers RUNX2 and BMP2 in vascular smooth muscle cells of the calcified aorta, while calponin and SM22 were increased. Additionally, allicin treatment increased GRP78, GRP94, and CHOP (endoplasmic reticulum stress markers), and prevented activation of the PERK/eIF2α/ATF4 signaling pathway, which is involved in apoptosis and osteoblast differentiation. Taken together, these effects contributed to preventing the progression of vascular calcification and aortic stiffness in an experimental model [65].

In short, allicin is anti-atherogenic throughout its antioxidant and anti-dyslipidemic effects with a plausible reduction in ischemic events. Furthermore, the evidence suggests that allicin can prevent vascular calcification and atherosclerosis, thus decreasing the risk of major adverse cardiovascular events and all-cause mortality.

### 3.3. Endothelial Dysfunction and Oxidative Stress

The endothelium is the inner cell lining of blood vessels (arteries, veins, and capillaries), thus it directly contacts the blood and circulating cells. The functions of the endothelium include the transport of nutrients and the regulation of vascular tone in response to vasoactive substances. However, the endothelium also has secretory capacities, thus it is considered an endocrine organ. Changes in blood components such as lipids, inflammatory cytokines, and free radicals directly target endothelial cells causing endothelial dysfunction. Moreover, it has been described that alterations in endothelial function may be a factor that triggers or contributes to CVD.

**Figure 4:**
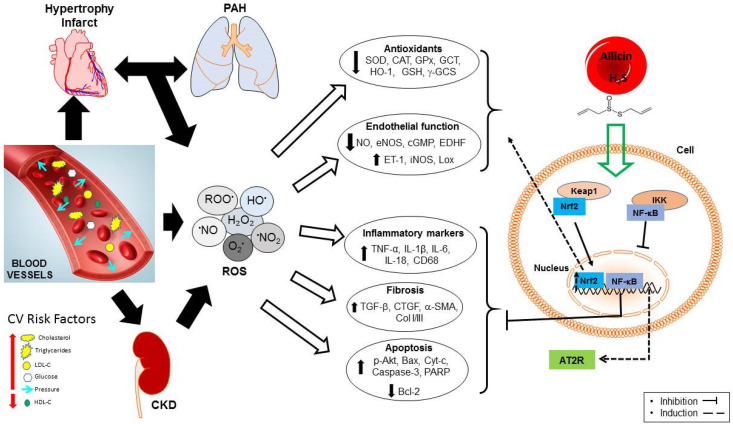
Allicin, through the stimulation of transcription factors, counteracts the deleterious effects of the imbalance between the formation and elimination of ROS in cardiovascular diseases. Abbreviations: angiotensin II receptor type 2 (AT2R); BCL2 associated apoptosis regulator (Bax); B-cell lymphoma 2 (Bcl-2); catalase (CAT); collagen I/III (Col I/III); connective tissue growth factor (CTGF); chronic kidney disease (CKD); cytochrome C (Cyt-c); cyclic guanosine monophosphate (cGMP); endothelin-1 (ET-1); endothelium-derived hyperpolarizing factor (EDFH); endothelial nitric oxide synthase (eNOS); glutathione peroxidase (GPx); γ-glutamyl cysteine synthase (γ-GCS); nuclear factor erythroid 2–related factor 2(Nrf2); heme oxygenase-1 (HO-1); high-density lipoprotein-cholesterol (HDL-C); hydrogen sulfide (H_2_S); endothelial nitric oxide synthase (eNOS); inducible nitric oxide synthase (iNOS); interleukin 1 beta (IL-1β); interleukin 6 (IL-6); interleukin 18 (IL-18); low-density lipoprotein-cholesterol (LDL-C); nitric oxide (NO); nuclear factor kappa B (NF-κB); oxidized lipids (Lox); poly(ADP-ribose) polymerase (PARP); pulmonary arterial hypertension (PAH); reactive oxygen species (ROS); reduced glutathione (GSH); superoxide dismutase (SOD); alpha-smooth muscle actin (α-SMA); transforming growth factor beta (TGF-β); tumor necrosis factor-alpha (TNF-α).

In this scenario, the imbalance between the formation and removal of ROS and free radicals are relevant features in the progression of CVD and could be caused by the interaction of risk factors such as dyslipidemia, insulin resistance, hypertension, and inflammation among others (Figure 4) [66]. Oxidative stress plays a key role in the development, maintenance, and progression of CVD. Oxidative stress decreases the bioavailability of NO and reduced glutathione (GSH), while it accelerates the oxidation of serum LDL-C and favors the lipid and protein oxidation in cells, which results in endothelial dysfunction, inflammation, fibrosis, and apoptosis (Figure 4) [66]. In addition, as described in the next sections, oxidative stress contributes to cell death during reoxygenation after ischemia. Hence, the modulation of oxidative stress is a useful maneuver in the treatment of CVD. Accordingly, in cell cultures, allicin protects cardiomyocytes and endothelial cells from apoptosis through inhibition of intracellular ROS production in a dose-dependent manner [67,68,69]. In endothelial cells and cardiomyocytes cultures, allicin reduced malondialdehyde (MDA) and increased NO release and endothelial NOS (eNOS) expression [70]. Moreover, it modulates the activity of caspase-3, NADPH oxidase (NOX), phase II detoxifying antioxidants enzymes, and protected from apoptosis through the regulation of poly(ADP-ribose) polymerase (PARP), pro-Caspase-3 and BCL2 associated X apoptosis regulator (Bax) [68,69,70]. Studies on healthy subjects reported that after two months of administration of garlic powder tablets (900 mg with contents of alliin and allicin at 1.3% and 0.6%, respectively), the GSH concentration increased in circulating erythrocytes [71]. In an experimental model of chronic kidney disease (CKD), allicin upregulated nuclear factor erythroid 2—related factor 2 (Nrf2), catalase (CAT), superoxide dismutase (SOD), and heme oxygenase-1 (HO-1), which was associated to decrements in oxidized proteins and lipid peroxidation in kidney tissue, improving the renal function [72].

On the other side, it has been described that endothelial dysfunction may be an early marker of atherosclerosis and a key factor in the progression of hypertrophy and heart failure. In primary cultures of endothelial progenitor cells (EPCs) obtained from CAD patients, it was observed that allicin increased EPCs proliferation in a dose-dependent manner, suggesting an improvement in endothelial function, even promoting neovascularization in patients with CAD [73]. In line with this, allicin may improve endothelial function in a dose-dependent manner through the increase in EPCs migration, these cells play a key role in neovasculogenesis and cardiac myocytes regeneration [11].

Altogether, these observations point out that allicin improves the total antioxidant status through the modulation of antioxidant enzymes and intracellular thiol pools reducing the deleterious effects of oxidative stress (inflammation and apoptosis), therefore leading to improvement in the endothelial function (Figure 4).

### 3.4. Myocardial Infarction

Myocardial infarction is produced by the arterial obstruction of one or more areas of the heart, subsequently resulting in a decrease or block in blood flow and oxygen supply, which causes the death of muscle cells and affects cardiac function. In line with this, in experimental myocardial ischemia-reperfusion (MI/R), allicin reduced myocardial injury and improved systolic and diastolic function in the heart. Other effects included decreased serum markers of heart damage, cardiac troponin, lactate dehydrogenase (LDH), creatine kinase (CK-MB), interleukin 6 (IL-6), IL-18, and tumor necrosis factor-alpha (TNF-α). Moreover, allicin provoked a reduction in lipid oxidation, while the activity of antioxidant enzymes (SOD, CAT, and glutathione peroxidase (GPx)) was shown to be increased [74,75]. Additionally, allicin reduced the infarct area, apoptotic index, and alleviated the pathological changes in the myocardium through the reduction in the phosphorylation of p38 and Bax. Concomitantly, the expression of the antiapoptotic B-cell lymphoma 2 (Bcl-2) protein was increased [74,75] On the other hand, using experimental models of ischemia in vivo and in vitro, allicin decreased intracellular calcium concentrations and Bax expression, in contrast, Bcl-2 was increased, resulting in the increase in cell viability and reduction in apoptotic index [76].

In hypoxia/reoxygenation conditions using cardiomyocyte cultures, allicin improved the cellular viability and reduced apoptosis, ROS generation, and the loss of mitochondrial membrane potential. Allicin downregulated the expression of proapoptotic Bax, cleaved caspase-3, cytochrome C (Cyt-c), IL-6, TNFα, hypoxia-inducible factor 1-alpha (HIF-1α), nuclear factor-kappa B (NF-κB), Nox4, protein kinase C (PKC), endothelin-1 (ET-1), and transforming growth factor-beta (TGF-β), while the expressions of Bcl-2, PGC1-α and eNOS were increased [77,78].

Other studies reported that allicin improved cardiac function, and reduced the myocardial infarct area, fibrosis, degeneration, necrosis and inflammatory cell infiltration in myocardial tissue as shown in experimental models of ischemia-reperfusion injury [77,79,80,81]. The cardioprotection induced by allicin was mediated by restoring the levels of SOD, CAT, GPx, and MDA in the myocardial tissue of rats [79]. Furthermore, in rats and mice with myocardial infarction allicin modulated the gene expression programs associated with the activation of signaling pathways involved in cell death and apoptosis by up-regulating the expression of eNOS, Bcl-2, phosphorylated phosphoinositide 3-kinase (p-PI3K), and phosphorylated protein kinase B (p-Akt) [77,79]. On the other hand, allicin down-regulated the expression of iNOS, p-eNOS/eNOS, Cyt-c, caspase-3, and caspase-9, Bax, phosphorylated Jun-amino-terminal kinase (p-JNK), phosphatidylinositol-4; 5-bisphosphate 3-Kinase catalytic subunit alpha PI3KCA(PI3K), and the phosphorylated G protein-coupled receptor kinase 2 (p-GRK2), Ca^2+^/calmodulin-dependent protein kinases (p-CaMKII), phospholipase C-gamma (p-PLC-γ), and inositol 1,4,5-triphosphate receptor (p-IP3R) [77,79]. Other studies in MI/R in mice showed that allicin, through modulation of the miR-19a-3p/PI3K/AKT pathway, can stimulate angiogenesis and thus revascularization of infarcted areas [81]. Moreover, it has been demonstrated that allicin improved coronary vascular reactivity in MI/R in rats through the production of hydrogen sulfide (H_2_S) and the regulation of Ca^2+^ homeostasis [80]. The beneficial effects of allicin on ischemia-reperfusion have been well-known and demonstrated using cell cultures as well as in vivo in experimental models.

The cardioprotective effects of allicin have also been evidenced at histologic, biochemical, molecular and functional levels and were exerted through its antioxidant, anti-inflammatory, antifibrotic, and antiapoptotic properties in the cardiotoxicity drug-induced, such as Trastuzumab- and Adriamycin-induced cardiotoxicity in rats [82,83]. Allicin was able to relieve the deleterious effects of reperfusion and it was mainly via modulation of oxidative stress, inflammation and apoptosis, which are common events during heart and kidney transplantation [84,85]. Noteworthy, the effects of allicin improved renal function and reduced the pathologic changes in renal tissue from rats with ischemia-reperfusion injury, while in cell cultures decreased apoptosis [84,85].

Thus, based on the clinic and experimental evidence, it can be summarized that allicin protects cardiac function and the cardiomyocytes against ischemia-reperfusion damage (myocardial infarction) mainly through the production of vasodilator substances and the modulation of pathways associated with apoptosis, oxidative stress, inflammation, and fibrosis (Figure 4).

### 3.5. Hypertension and Cardiac Hypertrophy

Hypertension is one of the main CVRF and the most common cause of cardiovascular morbidity and mortality. Because there are no easy-to-identify symptoms during hypertension, it is often called a “silent killer”, thereby people may have the problem without knowing it. At the onset of hypertension, the pressure inside the vessels (arteries) elevates over the physiological range, so the heart must work and pump harder against this pressure, causing hypertrophy of heart muscle (Figure 4) [86]. Therefore, cardiac hypertrophy is developed as a compensatory response to biomechanical stress (pressure and volume) and neurohormonal stimuli (angiotensin II, endothelin, and NO) (Figure 4). In patients with hypertension, left ventricular hypertrophy leads to heart failure and sudden death [86]. Moreover, hypertension also accelerates the development of atherosclerosis, systolic and diastolic dysfunction of the left ventricle, ischemic heart disease, and ventricular and supraventricular arrhythmia. Thus, the optimal control of blood pressure is a common therapeutic goal in the treatment of CVD.

In this respect, allicin induces antihypertensive effects in vivo in hypertensive rats, while in vitro showed concentration-dependent and endothelium-dependent vasorelaxant effects in vascular smooth muscle cells, such effects were mediated by the production of H_2_S. Allicin increased the levels of cyclic guanosine monophosphate (cGMP), cyclic adenosine monophosphate (cAMP), and endothelium-derived hyperpolarizing factor (EDHF)-mediated relaxation [87]. In hypertensive diabetic rats, allicin reduced hyperglycemia and hypertension by a mechanism related to the opening of an ATP-dependent potassium channel (K_ATP_ channel) with the participation of the type 2 sulfonylurea receptor (SUR2) [88]. On the other hand, in experimental CKD, allicin reduced hypertension, oxidative stress, and improved renal function. The anti-hypertensive effects of allicin were associated with the upregulation of angiotensin II receptor type 2 (AT2R) and eNOS as vasodilators [72]. In silico analysis suggested a potential site of interacting residues between allicin and the angiotensin II receptor type 1 (AT1R), which was similar to the interaction site between losartan and AT1R [72]. These data suggest that the antihypertensive effect of allicin could be partially mediated by the blockade of AT1R. Another study showed that allicin has similar therapeutic efficacy compared to the antihypertensive enalapril (an angiotensin-converting enzyme inhibitor) [36,37].

In hypertensive patients treated with allicin, a reduction in systolic and diastolic pressures after three and six months of allicin treatment was observed. However, the greatest antihypertensive effect was observed after six months of treatment [89]. Another study reported that the antihypertensive effect of allicin is observed between 5–14 h after the administration of a garlic preparation containing 1.3% allicin [90].

In cardiac hypertrophy established by abdominal aortic constriction (AAC) in rats, allicin improved cardiac function by inhibiting the hypertrophy through the inhibition of autophagy. The cellular mechanisms were dose-dependent and through phosphatidylinositol 3 kinase/serine threonine kinase/mammalian target of rapamycin (PI3K/Akt/mTOR) and mitogen-activated protein kinase/extracellular signal-regulated kinase/mTOR (MAPK/ERK/mTOR) axis [91]. Another study reported that in cardiac hypertrophy angiotensin-induced the cardioprotection of allicin was conferred by the suppression of oxidative stress, inflammation, and fibrosis. Moreover, allicin improved cardiac function, and systolic pressure, and prevented cardiac hypertrophy. The cellular mechanisms relied on the regulation of ERK1/2, JNK, AKT, NF-κB, and Smad 2/3 signaling pathways [92]. Other studies in rats with cardiac hypertrophy reported that the cardioprotective effects of allicin have been associated with the upregulation of the detoxifying enzymes regulated by the Nrf2/Keap 1 pathway (NADPH: quinone oxidase reductase 1 (NQO1), HO-1, γ-glutamyl cysteine synthase (γ-GCS), CAT, GPX, and SOD), and the inhibition of profibrotic proteins (collagen I/III) [93,94]. Additionally, the inhibitory effects of allicin on cardiac hypertrophy in rats can also be mediated by the inhibition of apoptosis and the stimulation of angiogenesis [95]. In cell cultures of cardiomyocyte allicin increase the expression of PPARα, PPARγ, and Bcl-2, and downregulate Bax, Fas, and Fax-L, these effects were induced by Angiotensin II stimulation and suggested that allicin can regulate apoptosis and ventricular remodeling [96].

In the case of pulmonary arterial hypertension (PAH), a progressive disease, it is thought to be a consequence of alterations in the function of the right ventricle (RV). In fact, the clinical outcome in PAH patients is determined by the response of RV to the increased afterload and is used as an indicator of PAH severity [97]. Thus, improving PAH will invariably impact RV structure and function. In this context, studies in experimental models of PAH characterized by pulmonary vascular remodeling, cardiac hypertrophy, and coronary endothelial dysfunction, have shown interesting data about the role of allicin on this subtype of CVD. Using an experimental model of PAH monocrotaline-induced (MCT) was observed that allicin improved the RV pressure and the function of the coronary endothelium PAH [98]. Another study reported that the allicin treatment prevented RV hypertrophy, pulmonary artery medial wall thickness, and fibrosis in the lungs. Furthermore, allicin decreased IL-1β, IL-6, TNF-α, Cd68, NF-κB, alpha-smooth muscle actin (α-SMA), and TGF-β in lungs and RV [99]. This suggests that through the improvement in coronary endothelial function, vasoreactivity, fibrosis, and inflammation allicin protects against cardiac hypertrophy in PAH. Thus, in PAH, allicin may be a therapeutic option to provide a better quality of life in patients through the improvement of heart and lung function which are closely related (Figure 4).

In summary, through its effects on blood pressure, oxidative stress, inflammation, autophagy, apoptosis, endothelial function, and ultimately through its favored activity on lungs, allicin provides protection in CVD from the deleterious effects of hypertension, cardiac hypertrophy, and PAH (Figure 4).

### 3.6. Diabetic Cardiomyopathy and Arrhythmias

Diabetes is one of the major causes of CVD and the leading cause of CKD worldwide. In fact, the CVD morbidity and mortality are 2–4 folds higher in patients with diabetes as compared with non-diabetic individuals [2]. Diabetic cardiomyopathy is characterized by alterations in cardiac structure (hypertrophy, fibrosis, remodeling, and apoptosis) and function, mainly mediated by hyperglycemia, hyperinsulinemia, and insulin resistance [100].

In experimental diabetes, allicin improved cardiac function and decreased hyperglycemia and intracellular calcium overload. Moreover, it protected the heart from diabetes-induced apoptosis through the increase in the expression of Bcl-2 and decrease in Fas. Furthermore, decreased fibrosis via the down-regulation of connective tissue growth factor (CTGF) and TGF-β in myocardial tissue [101]. In coronary artery endothelial cells obtained from healthy or diabetic donors, allicin stimulated NO production and eNOS expression [102]. This result suggests that allicin improved diabetes-induced coronary endothelial cell dysfunction.

The normal function of the heart is the contraction or beating, which occurs in response to electrical stimuli that control the atria and ventricles to contract in a synchronic and rhythmic manner. Any alterations in cardiac contractions are denominated arrhythmia and cause cerebral infarction and heart failure. Through in vitro and in vivo assays, it was shown that allicin regulates cardiac muscle contractions via modulation of the action potential and the expression of ion channels. The administration of allicin to diabetic rats delayed the onset of ventricular arrhythmia, reduced the score of arrhythmia, and shortened the action potential duration (APD) through inhibition of L-type calcium current (ICa-L) and enhancement of inward rectifier potassium current (IK1) [103]. Concurrently, in diabetic rats, the allicin treatment decreased the expression of L-type calcium channel protein (α1C) and increased the potassium channels protein (Kir2.1) mRNA in ventricular tissue [103]. In line with this study, in mouse ventricular myocytes, allicin gradually inhibited the transient outward potassium currents (I_to_) in a dose-dependent fashion. Furthermore, the high concentration of allicin accelerated the voltage-dependent inactivation of I_to_, which produced a significant effect on negative potential and cardiac repolarization [104]. In the primary cell culture of cardiomyocytes, allicin inhibited the translocation of the l-type calcium (Cav1.2) channel, from the endoplasmic reticulum to the cell membrane. In this system, Cav1.2 provides a pivotal substrate for cardiac electrophysiological activities [105]. The congenital long QT syndrome type 3 (LQT3) a hereditary arrhythmia is associated with mutations in the Nav1.5 channels, such as ∆KPQ. In cell culture, allicin reduced the late Na+ current (I_Na,L_) of the cardiac Na+ channel ∆KPQ-SCN5A, related to the dynamics of channel steady-state inactivation (SSI) and intermediate-state inactivation (ISI) by the drug, thus reducing the window current, which suggests that allicin may be useful in LQT3 therapy [106].

It is well known that loss or gain in ionic function channels in patients is associated with cardiac arrhythmias. In this manner, the results from experimental studies suggest that allicin may be useful in the treatment of arrhythmias, due to its effects on the expression and function of ionic channels.

## 4. Discussion

The treatment for CVD is mainly focused on controlling risk factors [12,13] and commonly includes pharmacological approaches and changes in lifestyle [14,15]. Until now, this type of intervention has been unsuccessful, because two or more drugs are required for optimal control of risk factors and usually have undesirable secondary effects. For this reason, the search for therapeutic options that contribute to preventing or delaying the progression of CVD continues.

CVD has a multifactorial origin, which makes its treatment difficult, thus the objective of the present study was to collect the available information on allicin and its beneficial effects on cardiovascular risk factors. In summary, data from experimental and clinical studies show that allicin decreases hypertension, inflammation, platelet aggregation, hyperglycemia, fibrosis, oxidative stress, and apoptosis and improves lipid profile, arrhythmias, and endothelial and cardiac function. For those reasons, and its low toxicity [25,26,56,90] allicin may be a reliable option for the treatment of the different clinic manifestations of CVD and its risk factors.

Allicin is an oily liquid, bright yellow in color, unstable, and with a short half-life [27]. However, due to its hydrophobic nature, it can be readily absorbed through cell membranes without inducing any damage to the phospholipid bilayer [107]. In this context, it has been reported that allicin is able to cross the erythrocyte cell membrane [35]. Possibly in this way, allicin can be distributed to various cell types, organs, and systems, including the CS to exert its beneficial effects. On a molecular level, the primary effect of allicin seems to be as an antioxidant and the multiple downstream effects described could be due to an indirect effect, since it can act as a bifunctional antioxidant. Due to its structure, allicin can act as a direct antioxidant by reacting directly with free radicals and ROS, acting as a substrate for glutathione synthesis, reacting with glutathione to produce S-Allyl-mercaptan glutathione, with L-cysteine to produce S-Allyl-mercaptan cysteine or other molecules that also can act as antioxidants [108,109]. On the other hand, allicin as an indirect antioxidant modulates the expression of cytoprotective genes. It is known that allicin reacts rapidly with the free thiol or sulfhydryl groups of the proteins in the cell membrane or in the different cell compartments [34,35]. It is likely that through this mechanism, allicin modifies proteins, second messengers, or transcription factors implicated in the regulation of gene expression. The regulation in the expression of antioxidant enzymes related to the Nrf2/Keap1 signaling pathway is possibly one of the well-known mechanisms through which allicin exerts antioxidant effects and has been considered an indirect mechanism [93,94,110,111]. Furthermore, it is tempting to hypothesize that the anti-inflammatory mechanisms of allicin related to the NF-κB/IκB-dependent pathway could be associated with activation or inhibition via the modification of sulfhydryl groups. However, further studies are required to confirm or discard the presence of sulfhydryl groups derivatives by allicin as the responsible mechanism involved in the regulation of cytoprotective gene expression. The antihypertensive effects of allicin may be exerted through the improvement of endothelial function and the modulation of proteins and substances associated with vasoactive responses (eNOS, angiotensin II receptors type 1 and type 2 (AT1R and AT2R), ET-1, cGMP, angiotensin, NO, and H2S). Through in vitro assays, it was demonstrated that allicin decreased the vascular reactivity to angiotensin II, and AT1R overexpression in the heart [94]. Another antihypertensive effect of allicin was demonstrated by its effects on the stimulation of the AT2R expression in renal tissue in CKD [72]. Moreover, the antioxidant effects of allicin could contribute to increasing the half-life of the vasoactive metabolites such as NO, and therefore to the maintenance of the vasodilatory actions. Additionally, the cardioprotective effects of allicin have been evidenced in the prevention of apoptosis of ischemic areas, as well as the induction of angiogenesis, which could be useful for the treatment of myocardial infarction, one of the first death causes worldwide [2,3,75,77,79,81]. The antiapoptotic and angiogenic activities of allicin may be useful to restore blood flow inducing recovery of injured areas and the effects on the modulation of expression and function of ion channels open the possibility of the use of allicin as an antiarrhythmic agent and pro-angiogenic.

A possible disadvantage of allicin is its poor stability and half-life. Thus, to improve the stability and half-life of allicin, a group of researchers conjugated allicin and captopril producing S-allylmercaptocaptopril (CPSSA). This compound was more stable and showed antihypertensive, lipid-lowering, and homocysteine-reducing effects, leading to the proposal of the use of this new conjugated molecule as a therapeutic option for the treatment of CVD [112,113]. Another study using an allicin-fenofibrate combination reported synergistic effects on the regulation of blood lipids and the improvement of vascular endothelial function [45]. For improving the stability and half-life of allicin, and for improving delivery and releasing, investigations have reported delivery systems including emulsion gels, nano emulsions, liposomes, microcapsules, and enteric-coated tablets [26,70,114,115].

Regarding the dose or concentration of allicin needed to obtain cardioprotective effects, allicin has demonstrated beneficial effects at low doses such as 8 mg/kg/day in experimental models [88]. The clinical studies have been carried out in patients with cardiovascular disease and healthy volunteers aged 20 years or older and the doses used a range from 10 to 48 mg/kg/day. No side effects, included bad smell, were reported in short-term or long-term studies [25,26,44,56,90]. However, healthy volunteers reported abdominal discomfort and stomachache subsequent to the administration of 48 mg of allicin once a day during dinner for 1 week on an empty stomach [63]. The intake of allicin from some garlic presentations has caused bad body odor and breath, possibly because quantities greater than one gram of garlic are necessary to obtain allicin in >0.6 mg [26]. To our knowledge, no study has used allicin in pediatric or children’s populations. A report assessed garlic extract therapy at 300 mg, 3 times a day/8 weeks, and no significant effect was observed on CVD risk factors in pediatric patients (8–18 years) with familial hyperlipidemia [116]. However, in adolescents, the garlic dry extract is recommended as a single dose (100–200 mg) once or twice a day, and the use in children under 12 years of age is not recommended [31]. Further studies are needed and will provide information about the safety, care, efficacy, and recommendations for the use of allicin in sensitive populations such as infants and children. Therefore, controlled clinical studies and trials with younger and pediatric patients are recommended to provide information about side effects or further data to support the use of allicin as a pharmacological option for the treatment of risk factors, pathogenic mechanisms, and clinical manifestations of CVD.

Garlic is a spice that has been attributed to beneficial effects on CV risk factors even since ancient times [30], but, so far, it is not used as a therapeutic option mainly because there are no standardized data on dosage, there are no specified garlic-derived compounds, or controlled studies on pharmacokinetics and pharmacodynamics, that allows replicating the beneficial effects. Recently, research has focused on studying the purified garlic compounds, showing important effects on various diseases [117,118]. Garlic has several sulfur compounds and depending on the species, maturation, processing method, or presentation, the quantity of one type of compound or another predominates [32]. In this context, allicin a garlic-derived compound has been isolated, purified or synthesized, quantified, characterized, and used in different dosages for the treatment of several cardiovascular diseases [26,107]. Despite the possible disadvantage of its poor stability [107] and half-life, the scientific literature provides convincing data that allicin alone or combined with anti-hypertensive [36,41,112,113] or anti-dyslipidemia drugs [45] may provide cardioprotective effects. In this sense, the use of delivery systems may be useful to increase absorption, bioavailability and efficacy, thus the biological activities and health benefits [26]. Allicin has shown beneficial effects on several metabolic alterations, risk factors, and cell injury. These effects have been observed in various organs, systems, and cell types (i.e., heart, endothelium, liver, and kidney). Therefore, this work provides a landscape of the scientific literature focusing on the beneficial effects of allicin on risk factors of CVD, as well as the cellular and molecular mechanisms involved. Various experimental and clinical studies used garlic instead of allicin and the therapeutic effects observed were attributed to allicin. However, patient data of scarce background, or studies involving garlic preparations from presentations (powder, aged, dry, black garlic, etc.) where allicin purity was not addressed, were not included in this review. Finally, in this review, the beneficial effects of allicin were examined, so this is intended to promote its use as an active ingredient, but not the use of garlic as a source of allicin. However, we consider important to describe the natural source of this interesting molecule.

## 5. Conclusions

CVDs are chronic, progressive, and have a multifactorial, and complex origin; therefore, more than one drug is required to achieve optimal control. Allicin exerts beneficial effects on various CVD risk factors, through the regulation of intracellular signaling pathways and mechanisms that improve cardiovascular structure and function, and in spite of the fact that few studies demonstrating its clinical utility have been reported, the evidence available to date suggests that allicin alone or in co-therapy may be an option in the treatment of CVD.

## Figures and Tables

**Table 1:** Classification of cardiovascular diseases based on the affected organ, system, or tissue.

Type	Affection	Clinical Manifestation
Cerebrovascular Disease	Alterations in blood vessels and circulation that supply blood to the brain [1,2,7]	Embolism
		Thrombosis
		Ischemic Stroke
		Intracerebral hemorrhage
		Transient ischemic attack
Coronary Heart Disease	Impaired flow in the blood vessels that supply blood to the heart [1,2,7]	Hypertensive diseases
		Myocardial infarction
		Heart failure
		Sudden death
		Atherosclerotic heart disease
		Pulmonary arterial hypertension
Peripheral Arterial Disease	The narrowing of the blood vessels reduces blood flow to the arms and legs [1,2,7]	Atherosclerosis
		Aneurysm
		Arterial thrombosis
		Deep vein thrombosis
		Acute limb ischemia
Arrhythmias	Alteration in rate or rhythm of the heartbeat [2,7]	Tachycardia
		Bradycardia
		Premature contractions
		Atrial fibrillation
Rheumatic Heart Disease	Damage to the muscle and valves in the heart [1,2,7]	Rheumatic fever
Congenital Heart Defects	Malformations of the heart or great vessels present at birth [2,9]	Abnormal heart valves
		Septal defects
		Patent ductus arteriosus
		Atresia
		Pulmonary arterial hypertension
		Coarctation of aorta

## Data Availability

Not applicable.
